# The effect of physical activities on internet addiction in college students: the mediating effect of self-control

**DOI:** 10.3389/fpsyg.2025.1530740

**Published:** 2025-02-05

**Authors:** Yan Sun, Yu Wang, Hongjun Yu, Jingmin Liu, Xiaolu Feng

**Affiliations:** ^1^Department of Physical Education, Beijing University of Posts and Telecommunications, Beijing, China; ^2^Department of Physical Education, University of Science and Technology Beijing, Beijing, China; ^3^Division of Sport Science and Physical Education, Tsinghua University, Beijing, China; ^4^College of Education, Zhejiang University, Hangzhou, Zhejiang, China

**Keywords:** internet addiction, self-control, physical activity, college student, exercise intensity, physical and mental health

## Abstract

**Objective:**

To investigate the relationship between college students' physical activities and Internet addiction, to investigate the role self-control control plays in this relationship, and to provide a theoretical foundation for the alleviation of college students' tendency to Internet addiction and intervention treatment.

**Methods:**

A questionnaire survey was conducted on 471 college students using the International Physical Activity Questionnaire (IPAQ), the Revised Chen Internet Addiction Scale (CIAS-R), and the Self-Control Scale (SCS).

**Results:**

Internet addiction was significantly negatively correlated with physical activities (overall; min/WK, *r* = −0.115, *P* < 0.05), with high-intensity physical activities (min/WK, *r* = −0.179, *P* < 0.01), and with low-intensity physical activities (*r* = −0.103, *P* < 0.05); self-control was significantly positively correlated with physical activities (overall; min/WK, *r* = 0.150, *P* < 0.01), with moderate—intensity physical activities (min/WK, *r* = 0.139, *P* < 0.01) while it was significantly negatively correlated with Internet addiction (min/WK, *r* = −0.349, *P* < 0.01). The mediating effect follows the path: physical activity → self-control → internet addiction.

**Conclusion:**

Physical activity can have a direct negative effect on college students' Internet addiction, and also influence Internet addiction through the mediating effect of self-control.

## 1 Introduction

At this juncture, the Internet penetration rate in China has attained 75.6%, with the proportion of Internet users accessing the Internet via mobile phones reaching an impressive 99.8% (China Internet Information Center, 2023). Mobile terminals have become an indispensable social life tool for the entire population. This phenomenon is especially evident among contemporary college students, who consider the internet to be an indispensable part of their lives. Concurrently, concerns regarding internet addiction among this demographic have escalated. The long-term use of the internet can easily exacerbate this tendency, leading to internet addiction or the potential for such a condition. The consequences of internet addiction among college students manifest at the individual level, affecting their physical and mental health, academic performance, career prospects, and familial relationships. At the macro level, these issues hinder China's strategic development through science and technology and talent, and hinder the construction of a modern socialist country.

The “Healthy China 2030” Plan Outline emphasizes that to foster the development of a healthy China, the principle of prioritizing prevention should be upheld, promote a healthy and civilized lifestyle, create a green and safe healthy environment, and reduce the occurrence of diseases. Excessive internet use during the college years can lead to increased sedentary time, leading to poor posture, prolonged sitting, lack of physical activity, and increased risk of obesity (Khan et al., 2017). Physical activity can ameliorate the condition of individuals with internet addiction (Liu, 2017; Li et al., 2020). Meanwhile, numerous studies have shown that inadequate self-control is associated with most problematic behaviors (Horn, 1995) and closely related to various behavioral addictions (Ayuningtyas, 2022; Arslan et al., 2021). Consequently, it can be hypothesized that self-control is a salient factor in the context of college students' internet addiction. In addition, experimental studies have shown that exercise can enhance individuals' executive functions, thereby boosting their self-control capabilities (Chang et al., 2012). Physical activity serves as a form of exercise that can augment individuals' self-control abilities (Susa et al., 2012). Given this, the possible mechanism of self-control between physical activity and internet addiction deserves further examination.

A divergence in the prevalence of Internet addiction among adolescents is observed according to differing parenting styles. Adolescents reared in an authoritarian milieu demonstrate comparatively superior performance with regard to Internet addiction, and the degree of parental supervision seems to inversely correlate with the likelihood of adolescents using video games to escape negative emotions (Commodari et al., 2024). This parenting style is believed to offer a degree of protection against Internet addiction in adolescents (Özgür, 2019), and it can be utilized as a protective factor against Internet addiction and online gambling addiction (Serna et al., 2023), fostering self-control and a sense of responsibility, thereby enabling adolescents to better cope with the temptations of the online world (Shimil and Srivastava, 2022). A study on early adolescents revealed that internet addiction is significantly and negatively correlated with positive reinforcement, warmth, supportiveness, and positive parenting styles, while it was positively and significantly associated with hostility, loose control, physical control, and broadband negative parenting styles (Erişim, 2020). The current study hypothesizes that authoritarian parenting styles may heighten the risk of internet addiction in adolescents (Shimil and Srivastava, 2022). The strictness imposed by authoritarian parents is more likely to lead adolescents to seek solace in the online world due to a lack of autonomous space and emotional support (Sarfika et al., 2023). A lack of opportunities for autonomous decision-making and self-management, stemming from prolonged strict parental control, may hinder the development of self-control (Yadav and Rupali, 2019; Fitriani, 2019). Conversely, laissez-faire parenting styles, characterized by minimal constraints and greater autonomy, have been associated with an elevated risk of Internet addiction in adolescents (Özgür, 2019; Sarfika et al., 2023; Niu et al., 2023). Moreover, it has been demonstrated that when parents adopt a permissive parenting style, their children are more likely to develop Internet addiction due to emotional problems such as worry (Lin et al., 2022). A robust correlation has been identified between parental education level and parenting style (Lin et al., 2022). Higher levels of maternal education have been demonstrated to be associated with reduced television screen time and computer use in children (Sanmarchi et al., 2021). Consequently, a balanced approach to parenting styles is imperative to mitigate the risk of internet addiction in adolescents. The educational level of family members is a factor that cannot be ignored when exploring the relationship between physical activity, self-control and Internet addiction.

In summary, this study is based on the reality that college students have a serious tendency toward internet addiction. Taking college students as the survey subjects, mathematical and statistical analysis methods are used to explore the correlation between physical activity and internet addiction, as well as to test the mediating effect of self-control, in order to provide reference for reducing internet addiction among college students.

## 2 Research trends and review

Through literature review, it has been found that since the 1990s, the issue of internet addiction among college students has garnered significant attention from scholars worldwide. The research scope includes multiple disciplines such as sociology, psychology, education, ideological and political education, and medicine. The main research topics related to this study involve the following aspects:

### 2.1 Concept of internet addiction

The academic community has not yet reached a consensus on the concept and understanding of internet addiction. Goldberg's seminal work pioneered the concept of internet addiction by drawing parallels with the definition of pathological gambling. He theorized that internet addiction constitutes an individual's impulsive and uncontrollable state of online behavior under the influence of non-addictive substances, and is a symptom of “coping mechanism behavior addiction.” This definition has been the subject of considerable controversy, particularly in the media, which has classified it as a “mental illness.” Since then, the American psychiatric community has carried out extensive research on internet addiction. Of particular note is the empirical research conducted by American psychologist Young, who posits that internet addiction should not be regarded as an independent mental illness, but rather as a manifestation of the existing “impulse control disorder” in internet users. This viewpoint aligns with the concept of “TV air-conditioning disease,” positing that it is merely a habitual psychological dependence arising from prolonged exposure, thereby categorizing it as a psychological problem, specifically behavioral dependence. Armstrong's theory posits that individuals' propensities for internet usage are influenced by their unique personality traits, which, in turn, are shaped by the functional characteristics of the internet platforms they frequent. This theory predicts the emergence of diverse types of internet usage, ranging from the pure to the mixed. A patient suffering from internet overuse can be a pure type or a mixture of several types, and in reality, the majority of patients are mixed type. According to the clinical manifestations, internet addiction is systematically divided into five types: internet addiction, internet relationship addiction, internet compulsive behavior, information collection addiction, and computer addiction (Chaomin, 2012).

In China, the “Clinical Diagnostic Criteria for Internet Addiction” formulated by Tao Ran from the Beijing Military Region General Hospital in 2008 has attracted attention from all sectors of society. Internet addiction refers to a psychological and behavioral disorder caused by an individual's repeated excessive use of the Internet, which is divided into five categories: addiction to online games, addiction to online pornography, addiction to online relationships, addiction to online information, and addiction to online transactions. Among them, online game addiction is the most common, accounting for 82%. In 2009, the Ministry of Health of China rejected the use of internet addiction as a clinical diagnosis of mental illness when soliciting opinions on the “Guidelines for the Healthy Use of the Internet by Minors,” stating that the current definition of internet addiction is inaccurate and should not be used to define the harm caused by improper use of the internet to human health and social function. it is posited that internet addiction is solely attributed to the improper use of the internet (Disease Control Prevention Bureau of the Ministry of Health, 2023). In the 11th edition of the International Classification of Diseases in 2018, the World Health Organization added “gaming addiction” to ICD-11 and listed it as a mental illness. The Core Information and Interpretation of Health Education for Chinese Teenagers (2018 Edition) (National Health Commission, 2018) released by the National Health Commission clearly defines the definition and diagnostic criteria of Internet addiction, and believes that Internet addiction refers to the uncontrolled behavior of Internet use impulse without the effect of addictive substances, which is manifested in the obvious academic, occupational and social function damage caused by excessive use of the Internet. a key criterion for diagnosing internet addiction disorder is that the individual's relevant behaviors must persist for at least 12 months. To sum up, Internet addiction can be understood as the phenomenon of a series of psychological, physical and mental problems caused by individuals' improper use of the Internet for a long time in addition to work and study.

### 2.2 Diagnostic criteria and measurement of internet addiction

Foreign scholars were the first to pay attention to and study the phenomenon of internet addiction, and have developed a large number of psychometric internet addiction scales. As early as Young proposed the Internet Addiction Diagnostic Questionnaire based on the American Psychiatric Classification and Diagnostic Manual (DSM-V), which mainly judges the degree of Internet addiction from aspects such as internet use function, emotional involvement, irrational cognition, and personal life events. If participants give positive answers to five out of eight questions, it is determined to be Internet addiction (Young, 2009); the significance of Morahan-Martin and Schumacher's (2000) “Pathological Internet Use Questionnaire” is in differentiating the psychological criteria of high engagement and addiction; Davis et al.'s “Online Cognitive Scale” includes four subscales: social comfort, loneliness and depression, reduced impulse control, and attentional shift, with a total of 36 questions. It belongs to a 7-level self-report scale (1 = least agree, 7 = most agree), with higher scores indicating deeper levels of internet addiction. However, “subjects are not clear about what to measure” and “items measure subjects' cognition rather than behavior” make this scale have advantages that other internet addiction scales do not have (Davis et al., 2002); Scott and Caplan's “Revised Generalized Problem Internet Usage Scale” includes five dimensions: online social performance, emotional regulation, cognitive involvement, network use compulsion, and negative consequences. Seven point scoring (1 = very disagree, 7 = very agree). The higher the score, the more severe the internet addiction issue. The scale found that the lack of self-regulation was an important factor in the negative consequences of individual Internet use (Caplan, 2002).

Chinese scholars have conducted surveys and revisions on the above-mentioned scales, and some scholars have also applied the English version of the scales in psychological research on Chinese students (Dongmei et al., 2017). In China, the development of Internet addiction related research can be divided into two stages: 2001–2008 and 2009 to the present. There are also many scales and standards for Internet addiction, among which the Chinese Internet Addiction Scale Revised (CIAS-R) prepared by Chen Shuhui, the Chinese Internet Addiction Scale Revised by Young, and the Adolescent Pathological Internet Usage Scale (APIUS) prepared by Li and Yang (2007) are widely used. Through literature review, it can also be found that scholars have developed individual case internet addiction survey questionnaires for college students in certain regions, and the design of each scale has its own characteristics. The characteristics of different scales also determine the measurement scales and research outcomes.

### 2.3 Research on physical activity and internet addiction

Based on the four elements of physical activity: frequency, intensity, time, and type, the research related to internet addiction is summarized as follows: Kujach et al. (2018) studied the effects of high-intensity interval exercise and dorsolateral prefrontal activity, and found that short-term high-intensity exercise can increase the release levels of neurotransmitters and BDNF in the brain, effectively improving cognitive performance; Fan et al. (2021) conducted a study on the influencing factors of low, medium, and high exercise intensities on college students' use of smartphones, and found that after 30 min of acute aerobic exercise, the accuracy of non target stimuli significantly improved, while the accuracy of medium intensity non target stimuli changed the most; Yang et al. (2024) found that moderate intensity acute aerobic exercise can effectively promote inhibitory function by allocating cognitive resources more reasonably when exploring the dose relationship between exercise intensity and inhibitory function. The research findings of the aforementioned scholars indicate that exercise of moderate and higher intensity can effectively enhance cognitive performance, improve participants' inhibitory control, and thus inhibit psychological processing and behavior unrelated to the current goal, such as having a withdrawal effect on internet addiction. In terms of team sports in ball games, Li et al. (2011), Yinghai (2013), Yunsheng and Yang (2016), and Xiao et al. (2021) conducted intervention studies on adolescents and college students for 8–16 weeks, 3–5 times a week, and 35–150 min per session, respectively. They found that exercise has a good effect on improving mental health and quitting internet addiction, especially on reducing mild and moderate internet addiction. In terms of research on joint intervention of exercise and psychology, Liu (2017), Hong et al. (2020), Zhao et al. (2021) conducted a 6–12 week study on adolescents and college students, with 1–2 times a week, 30–60 min/time, 60%−70% exercise intervention and 1–2 times a week, 60–120 min/time psychological therapy intervention. They found that joint intervention significantly enhanced the activation level of the prefrontal cortex, improved psychological craving and symptoms, and is an effective means of quitting internet addiction.

More studies have shown that exercise can improve GSI, depression, anxiety, aggression, somatization, social insecurity, fear and anxiety, paranoid ideation, and mental illness (Liu et al., 2017), leading to positive psychological feelings, enhanced physical fitness, improved self-evaluation, increased willpower, and self-control (Ziliang and Wenkai, 2002). However, attention should be paid to important moderating variables such as the dosage of exercise intensity, diversity of exercise styles, and exercise duration. At the same time, attention should also be paid to the combined intervention of physical activity and psychological therapy. At present, there are relatively few studies on the relationship between physical activity and internet addiction both domestically and internationally, and research on the underlying mechanisms of the impact of physical activity on internet addiction is particularly scarce. Therefore, researchers should combine psychological research methods, such as using psychological scales, to explore the relationship between the two from more perspectives.

## 3 Research methods

### 3.1 Literature review method

Using keywords like “physical activity,” “sports activity,” “internet addiction,” and “self-control,” searches were conducted in Chinese databases such as CNKI Journal Database and Baidu Scholar. Using keywords such as “Sport and Exercise Psychology,” “Internet addiction,” and “Self Control,” research literature on the relationship between physical activity and internet addiction among foreign university students was collected in foreign databases such as Web of Science and EBSCO. As of August 2023, 1,420 relevant academic papers were identified, and 322 core journal papers focusing primarily on “college students' internet addiction” were retrieved. After further investigation, it was found that there are 51 literature closely related to this study.

### 3.2 Questionnaire survey method

#### 3.2.1 Questionnaire design

The first survey questionnaire is the short form of the International Physical Activity Questionnaire (IPAQ). The scale is a commonly used questionnaire survey tool. It was developed in Geneva in 1998. So far, it has passed a large number of reliability and validity tests in many countries or regions around the world. At present, it has been used in Chinese population research, and has been tested to have good reliability and validity. This questionnaire has the characteristics of “simple operation, short time consumption, and detailed,” and is suitable for large-scale testing of the 18-year-old population. The International Physical Activity Questionnaire (IPAQ) short questionnaire consists of seven questions, asking the sample individuals about their physical activity types (step type = 3.3 METs, moderate intensity activity = 4.0 METs, vigorous intensity activity = 8.0 METs), frequency and duration, as well as their sleep status over the past 7 days (Macfarlane et al., 2007). Based on the answer, the MET value for the type of physical activity can be calculated. MET value is a unit representing metabolic equivalent and is a method used to measure physical activity intensity, where one MET equals the rate of energy expenditure in a static or resting state, typically 3.5 ml of oxygen per kilogram per minute. Depending on the nature and intensity of the activity, the MET value will also vary. For example, if the physical activity is playing table tennis, then find a specific MET value of four in the physical activity outline. Therefore, by calculating the MET value corresponding to physical activity in IPAQ, one can understand the intensity and type of physical activity that a person has participated in in the past 7 days.

The second survey questionnaire is the Chinese version of the Revised Chen Internet Addiction Scale (CIAS-R) in Chinese. This scale was developed by Professor Chen Shuhui from Taiwan in 1999, using college students as a sample, based on the diagnostic criteria of DSM-IV for various addiction symptoms. It consists of 26 questions and adopts the Likert 4-point scoring method, including five dimensions: obsessive-compulsive symptoms, withdrawal symptoms, tolerance symptoms, interpersonal health problems, and time management problems. The total score of the full scale represents the degree of individual internet addiction, and the higher the total score, the higher the tendency toward internet addiction (potential internet addicts ≥58 points, internet addicts ≥68 points).

The third survey questionnaire is the Chinese version of the self-control scale (SCS). The scale was developed and published by Tangney in the United States in 2004, and later translated, validated, and revised by Unger et al. (2016), confirming that the scale can be used in China. After revision, the scale retained the 36 questions in the English version and was scored on a 5-point scale (1 = strongly disagree, 5 = strongly agree). It includes five dimensions: general ability of self-discipline, thoughtful consideration, healthy habits, professional ethics, and reliability. Among them, questions 1, 5, 7, 13, 15, 18, 22, 26, 27, and 36 are scored positively, while the rest of the questions are scored negatively. The higher the total score, the better the self-control ability.

#### 3.2.2 Reliability and validity test of questionnaire

The reliability and validity of the Chinese version of the International Physical Activity Questionnaire (IPAQ) Short Form for college students are equal to or higher than those of similar questionnaires (IPAQ) Short Form among college students are higher than or equal to similar questionnaires, and the correlation coefficients within each physical activity group are all above 0.7, indicating high reliability and validity (Macfarlane et al., 2007). It is suitable for this study.

Chinese Internet Addiction Scale: The test-retest reliability of the selected Chinese Internet Addiction Scale (CIAS-R) is 0.83, and the internal consistency coefficients between each subscale are satisfactory, ranging from 0.79 to 0.89. The total Cronbach's alpha of the scale is 0.976. In this study, Cronbach's alpha was used to measure the intrinsic reliability of the questionnaire, with a reliability coefficient value of 0.955. Before the formal survey, the reliability of the pre-survey can be tested by using the CITC and the alpha coefficient of the deleted items, and for the 'alpha coefficient of the deleted items', after the deletion of any question item, the reliability coefficient does not increase significantly. As for the “CITC value,” the CITC values of the analyzed items are all >0.4, which indicates that there is a good correlation between the analyzed items, and at the same time it also indicates that the reliability level is good. In conclusion, the reliability coefficient value of the research data is higher than 0.9. Validity has been checked using KMO and Bartlett's test, and from the above table it can be seen that: the KMO value is 0.959, which is >0.8, and the above data comprehensively indicate that the quality of reliability and validity of the data is applicable to this research.

Self Control Scale: the Chinese version of the Self Control Scale (SCS) selected in this article has a total Cronbach's alpha of 0.88 and an alpha range of 0.58–0.81 for each dimension (Unger et al., 2016). Cronbach's alpha was used in this study to measure the intrinsic reliability of the questionnaire. The reliability coefficient value is 0.855, which is >0.8, indicating the high quality of reliability of the study data. For the “alpha coefficient for deleted items,” the reliability coefficient does not increase significantly when a question item is deleted, indicating that the question item should not be deleted from the questionnaire. KMO and Bartlett's test were used to check the validity, as can be seen from the table above: the KMO value is 0.903, the KMO value is more than 0.8, the research data is very suitable for extracting information, which means it is suitable for this study.

#### 3.2.3 Research objective

A review of the extant literature on sample sizes utilized in analogous studies, coupled with a stratified sampling procedure, was conducted in conjunction with the number of students enrolled in the spring 2023 semester and voluntary participation. A random sampling procedure was conducted on a class-wide basis in April 2023, encompassing indicators for grade level (freshman, sophomore, junior) and gender (male, female). The rationale behind this approach is to establish a certain degree of homogeneity and stability within the class, which is conducive to the control of variables in the study and the enhancement of its accuracy and reliability. According to the teaching requirements of physical education courses at Beijing University of Posts and Telecommunications (BUPT), a signing-in system is adopted, and the questionnaires are distributed and recovered through questionnaire stars in the theoretical teaching part of the physical education courses. The students fill out the questionnaires before they fill out the other questionnaires. The respondents were informed by the teachers of the objective of the survey and that participation was voluntary. A total of 485 questionnaires were distributed. Subsequent to distribution, the data collection, entry, cleaning and analysis were meticulously reviewed to ensure the accuracy and completeness of the data. The final valid questionnaires were 471, with a validity rate of 97.1 per cent.

### 3.3 Mathematical and statistical methods

After standardizing the variables, descriptive statistics, sample mean *t*-test, one-way ANOVA, correlation analysis, and multiple regression analysis were conducted on the physical activity level, internet addiction, and self-control of college students using statistical analysis software SPSS25.0 after normality testing. By employing the Bootstrap method in the Process macro program in conjunction with the stepwise test method, model 4 is utilized to analyze the mediating effect of self-control.

## 4 Results and analysis

### 4.1 Basic information of the investigated subject

The age range of the survey subjects is 17–26 years old, including 132 females, accounting for 28.03%, and 339 males, accounting for 71.97%. The number of males significantly exceeds that of females. This discrepancy in the gender ratio may potentially influence the study's outcomes, particularly in regard to perceptions and behaviors concerning specific issues, which may vary according to gender. There are 196 students from the eastern region, accounting for 41.61%, 160 students from the central region, accounting for 33.97%, and 115 students from the western region, accounting for 24.42%. Such regional distribution reflects a certain degree of diversity. Students in different regions may be influenced by different social environments, educational resources and cultural traditions, thus showing differences in self-control, physical activity habits and the risk of Internet addiction. In terms of grade distribution, freshmen are the main group, with 311 students, accounting for 66.03%, followed by sophomores with 107 students, accounting for 22.72%, and juniors with 53 students, accounting for 11.25%. It is evident that variances exist with regard to the learning experience and social exposure of students across different grades. These differences may potentially affect their perceptions and behaviors (Table 1).

**Table 1:** Basic information of the subject under investigation.

**Category**		**Number of people**	**Proportion (%)**	**Mean ±standard deviation**
Gender	Male	339	71.97	–
	Female	132	28.03	–
Age	17	3	0.64	19.18 ± 1.128
	18	140	29.72	
	19	174	36.94	
	20	100	21.23	
	21	39	8.28	
	22	11	2.34	
	23	2	0.42	
	24	1	0.21	
	26	1	0.21	
Hometown area	Eastern region	196	41.61	–
	Central region	160	33.97	–
	Western region	115	24.42	–
Grade	Freshman	311	66.03	–
	Sophomore	107	22.72	–
	Junior	53	11.25	–
Height	Male	339	71.97	1.766 ± 0.063
	Female	132	28.03	1.646 ± 0.057
Weight	Male	339	71.97	70.886 ± 12.792
	Female	132	28.03	55.223 ± 7.433
BMI	Male	339	71.97	22.704 ± 3.729
	Female	132	28.03	20.342 ± 2.239
Total		471	100	

### 4.2 Score of college students' internet addiction test questionnaire

From Table 2, it can be seen that the number of individuals with high immunity is relatively small, with only one person, while the number of generally immunized individuals is 55, accounting for 11.68%, number of college students potentially with internet addiction accounts for 51.59%, and the number of internet addicts accounts for 36.52%.

**Table 2:** Scores of college students' internet addiction test questionnaire.

**Name**	**Option**	**Number of people**	**Percentage (%)**
Total score of internet addiction	High immunity	1	0.21
	General immunity	55	11.68
	Potential internet addiction	243	51.59
	Internet addiction	172	36.52
Total		471	100

### 4.3 The relationship between physical activity, internet addiction, and self-control among college students

As shown in Table 3, it can be seen that descriptive statistics and independent sample *t*-test were used to analyze the physical activity levels of college students at different intensities. The results show that male and female students exhibited a significant 5% level of min/WK for high-intensity physical activity, and the specific comparisons reveal that the average value for male students was 82.04, significantly higher than the average value for female students at 65.83. There is a significant difference of 1% between males and females in terms of low-intensity physical activity, and the specific comparison shows that the average value for males is 141.45, which is significantly lower than the average value for females, which is 178.75. There is a significant difference of 5% in physical activity intensity (total) between males and females, and the specific comparison shows that the average value for males is 474.09, which is significantly lower than the average value for females of 551.55. There is no significant difference between males and females in terms of moderate intensity physical activity.

**Table 3:** Descriptive statistics and correlation analysis of physical activity, internet addiction, and self-control among college students.

	**Male (mean ±standard deviation)**	**Female students (mean ±standard deviation)**	***T* statistic**	**Total score of internet addiction**	**Total self-control score**
High intensity physical activity min/WK	82.04 ± 72.86	65.83 ± 68.85	2.201^*^	−0.179^**^	0.089
Moderate intensity physical activity min/WK	62.65 ± 74.77	76.74 ± 77.96	−1.815	−0.064	0.139^**^
Low intensity physical activity min/WK	141.45 ± 112.19	178.75 ± 119.09	−3.185^**^	−0.103^*^	0.078
Physical activity intensity (total) min/WK	474.09 ± 386.01	551.55 ± 374.94	−1.972^*^	−0.115^*^	0.150^**^

* *p* < 0.05;

** *p* < 0.01.

Using correlation analysis to study the correlation between the total score of internet addiction and four items: high-intensity physical activity, moderate intensity physical activity, low-intensity physical activity, and total physical activity intensity. The results show that the correlation coefficient between the total score of internet addiction and high-intensity physical activity was −0.179, with a significance level of 1%, indicating a significant negative correlation between the total score of internet addiction and high-intensity physical activity. The correlation coefficient between the total score of internet addiction and low-intensity physical activity is −0.103, and shows a significance level of 5%, indicating a significant negative correlation between the total score of internet addiction and low-intensity physical activity. The correlation coefficient between the total score of internet addiction and physical activity intensity (total) is −0.115, and shows a significance level of 5%, indicating a significant negative correlation between the total score of internet addiction and physical activity intensity (total).

Using correlation analysis to study the correlation between the total score of self-control and four items: high-intensity physical activity, moderate intensity physical activity, low-intensity physical activity, and overall physical activity intensity. The results show that the correlation coefficient between the total score of self-control and moderate intensity physical activity was 0.139, with a significance level of 1%, indicating a significant positive correlation between the total score of self-control and moderate intensity physical activity. The correlation coefficient between the total score of self-control and physical activity intensity (total) is 0.150, and shows a significance level of 1%, indicating a significant positive correlation between the total score of self-control and physical activity intensity (total).

### 4.4 The relationship between self-control and internet addiction among college students

As indicated in Table 4, Pearson correlation analysis was used to study the relationship between self-control and internet addiction among college students. The results show that the correlation coefficient between the total score of self-control and internet addiction was −0.349, which passed the 1% significance level test, indicating a significant negative correlation between self-control and internet addiction.

**Table 4:** Correlation coefficient between self–control and internet addiction among college students.

	**Total score of internet addiction**	**Total self–control score**
Total score of internet addiction	1	
Total self–control score	−0.349^**^	1

** *p* < 0.01.

### 4.5 Gradual regression analysis of the relationship between physical activity and internet addiction among college students

A stepwise regression analysis was performed, with high-intensity physical activity, moderate-intensity physical activity, and low-intensity physical activity serving as independent variables, gender, hometown area, family income, father's education level, and mother's education level as covariates, and the total score of internet addiction as the dependent variable. Following automatic recognition by the model, a total of two items remained in the model, namely “mother's education level” and “high-intensity physical activity.” The mathematical expression of the model is as follows:


(1) $$\begin{array}{c} Y=\text{65.125}-1.776\times X_{8}-\text{0.033}\times X_{1} \end{array}$$

The explanatory coefficient (*R*^2^) of the relational model is 0.049, and the adjusted explanatory coefficient is 0.045. The *F*-statistic of the model is 11.971, which passed the 5% significance level test, indicating the effectiveness of the model. Furthermore, an investigation into the multicollinearity of the model revealed that all VIF values were <5, indicating the absence of collinearity issues. Additionally, the *D–W* value was found to be ~2, suggesting that the model does not exhibit autocorrelation and there is no correlation between the sample data. The model demonstrates adequate performance and possesses considerable predictive capability. The regression coefficients of the relationship model further substantiate this claim, with maternal education level and high-intensity physical activity exerting a significant negative influence on college students' internet addiction, as evidenced by regression coefficients of −2.873 and −3.726, respectively. This finding suggests that the influence of maternal education level and high-intensity physical activity on college students' internet addiction is more significant, while the influence of other independent and covariates is weaker (Table 5).

**Table 5:** Stepwise regression analysis of the relationship between physical activity and internet addiction in college students.

	**Non standardized coefficient**		**Standardization coefficient**	***T* statistic**
	**Regression coefficient**	**Standard deviation**		
Constant	65.125	1.991	–	32.718
Mother's education level (X8)	−1.776	0.618	−0.13	−2.873^**^
High intensity physical activity min/WK (X1)	−0.033	0.009	−0.169	−3.726^**^
*R* ^2^	0.049			
Adjust *R*^2^	0.045			
*F*	*F*_(2,468)_ = 11.971, *p* = 0.000			
*D*–*W* value	2.01			

^*^p < 0.05;

** *p* < 0.01.

### 4.6 Mediating effect test of self-control between physical activity and internet addiction

It is evident that the mediation effect analysis involves three distinct models.

As demonstrated in Table 6. The mathematical expression for Equation 1 is:


(2) $$\begin{array}{c} Y_{1}=61.383+2.382\times X_{1}+0.334\times X_{2}+0.263\times X_{3}-0.860\\ \times X_{4}-1.451\times X_{5}-0.004\times X_{6} \end{array}$$

**Table 6:** Regression analysis of mediation model–*XY* total effect equation (*n* = 471).

	**Non standardized coefficient**		**Standardization coefficient**	***t***	***p***
	***B***	**Standard deviation**	**Beta**		
Constant (C)	61.383	3.366	–	18.235	0.000^**^
Gender (X1)	2.382	1.425	0.077	1.672	0.095
Hometown location (X2)	0.334	0.809	0.019	0.413	0.68
Family income (X3)	0.263	0.411	0.033	0.639	0.523
Father's education level (X4)	−0.86	0.925	−0.059	−0.929	0.353
Mother's education level (X5)	−1.451	0.852	−0.106	−1.704	0.089
Physical activity intensity (total) min/WK (X6)	−0.004	0.002	−0.107	−2.321	0.021^*^
*R* ^2^	0.038				
Adjust *R*^2^	0.026				
*F*	*F*_(6,464)_ = 3.074, *p* = 0.006				
*D*–*W* value	2.024				

The dependent variable in this study is the total internet addiction score.

* *p* < 0.05;

** *p* < 0.01.

The coefficient of determination (*R*^2^) of the model is 0.038, the adjusted explanatory coefficient is 0.026, and the *F*-statistic of the model is 3.074. The model has passed the test at the 5% significance level, indicating that Equation 1 is effective. This means that at least one of the following factors will have an impact on the total score of internet addiction: gender, hometown, household income, father's education level, mother's education level, and activity intensity (total). Furthermore, an examination of the multicollinearity of the model revealed that all VIF values were <5, indicating the absence of collinearity issues. Furthermore, the *D*–*W* values were found to be ~2, suggesting that the model did not exhibit autocorrelation and that there was an absence of correlation between the sample data. The model demonstrates adequate predictive capability. The regression coefficient of the relationship model indicates that physical activity intensity (total) has a significant negative impact on the total score of internet addiction, while gender, hometown, family income, father's education level, and mother's education level do not have an impact on the total score of internet addiction.

As demonstrated in Table 7. The mathematical expression for Equation 2 is:


(3) $$\begin{array}{c} Y_{2}=107.352-2.046\times X_{1}-1.249\times X_{2}-0.210\times X_{3}+1.455\\ \times X_{4}+1.727\times X_{5}+0.006\times X_{6} \end{array}$$

**Table 7:** Regression analysis of mediation model–*XM* total effect equation (*n* = 471).

	**Non standardized coefficient**		**Standardization coefficient**	***t***	***p***
	***B***	**Standard deviation**	**Beta**		
Constant (C)	107.352	3.779	–	28.408	0.000^**^
Gender (X1)	−2.046	1.6	−0.058	−1.279	0.202
Hometown location (X2)	−1.249	0.908	−0.063	−1.375	0.17
Family income (X3)	−0.21	0.462	−0.023	−0.454	0.65
Father's education level (X4)	1.455	1.039	0.088	1.401	0.162
Mother's education level (X5)	1.727	0.956	0.111	1.806	0.072
Physical activity intensity (total) min/WK (X6)	0.006	0.002	0.137	3	0.003^**^
*R* ^2^	0.06				
Adjust *R*^2^	0.048				
*F*	*F*_(6,464)_ = 4.942, *p* = 0.000				
*D–W* value	1.77				

The dependent variable in this study is the total score on the self–control scale.

** *p* < 0.01.

The explanatory coefficient (*R*^2^) of the relationship model is 0.060, the adjusted explanatory coefficient is 0.048, and the *F*-statistic of the model is 4.942. The model has passed the test at the 5% significance level, indicating that Equation 2 is effective. This means that at least one of the following factors will have an impact on the total self-control score: gender, hometown, household income, father's education level, mother's education level, and physical activity intensity (total). Furthermore, an investigation into the multicollinearity of the model revealed that the VIF values were all <5, indicating that there was no collinearity issue, and the *D*–*W* values were around the number 2, indicating that the model did not have autocorrelation and there was no correlation between the sample data. The model demonstrates efficacy and exhibits robust predictive capabilities. The regression coefficient of the model indicates that physical activity intensity (total) has a significant positive impact on the total score of self-control. However, the impact of gender, hometown location, family income, father's education level, and mother's education level on the total score of internet addiction is not significant.

As demonstrated in Table 8. The mathematical expression for Equation 3 is:


(4) $$\begin{array}{c} Y_{1}=91.782+1.803\times X_{1}-0.019\times X_{2}+0.203\times X_{3}-0.448\\ \times X_{4}-0.962\times X_{5}-0.002\times X_{6}-0.283\times X_{7} \end{array}$$

**Table 8:** Regression analysis of mediation model–the following equation is a direct effect equation for *X* (*n* = 471).

	**Non standardized coefficient**		**Standardization coefficient**	***t***	***p***
	***B***	**Standard deviation**	**Beta**		
Constant (C)	91.782	5.288	–	17.357	0.000^**^
Gender (X1)	1.803	1.355	0.058	1.331	0.184
Hometown location (X2)	−0.019	0.769	−0.001	−0.025	0.98
Family income (X3)	0.203	0.39	0.026	0.521	0.603
Father's education level (X4)	−0.448	0.88	−0.031	−0.509	0.611
Mother's education level (X5)	−0.962	0.811	−0.07	−1.186	0.236
Physical activity intensity (total) min/WK (X6)	−0.002	0.002	−0.063	−1.426	0.154
Self–control total score	−0.283	0.039	−0.322	−7.214	0.000^**^
*R* ^2^	0.135				
Adjust *R*^2^	0.122				
*F*	*F*_(7,463)_ = 10.360, *p* = 0.000				
*D*–*W* value	2.017				

The dependent variable in this study is the total score on the self–control scale.

** *p* < 0.01.

The explanatory coefficient (*R*^2^) of the relationship model is 0.135, the adjusted explanatory coefficient is 0.122, and the *F*-statistic of the model is 10.360. The model has passed the test at the 5% significance level, indicating its effectiveness. That is to say, at least one of the following factors has a significant impact on the total score of internet addiction: gender, hometown area, household income, father's education level, mother's education level, physical activity intensity (total), and self-control total score. In addition, an investigation into the multicollinearity of the model revealed that all VIF values are <5, indicating the absence of collinearity. Furthermore, the *D*–*W* value is ~2, suggesting that the model does not exhibit autocorrelation and there is no correlation between the sample data. This affirms the model's efficacy and strengthens its predictive capacity. The regression coefficient of the model indicates that self-control exerts a significant negative influence on the total score of internet addiction. However, gender, hometown location, family income, father's education level, mother's education level, and overall physical activity intensity do not have a significant impact on the total score of internet addiction.

**Figure 1:**
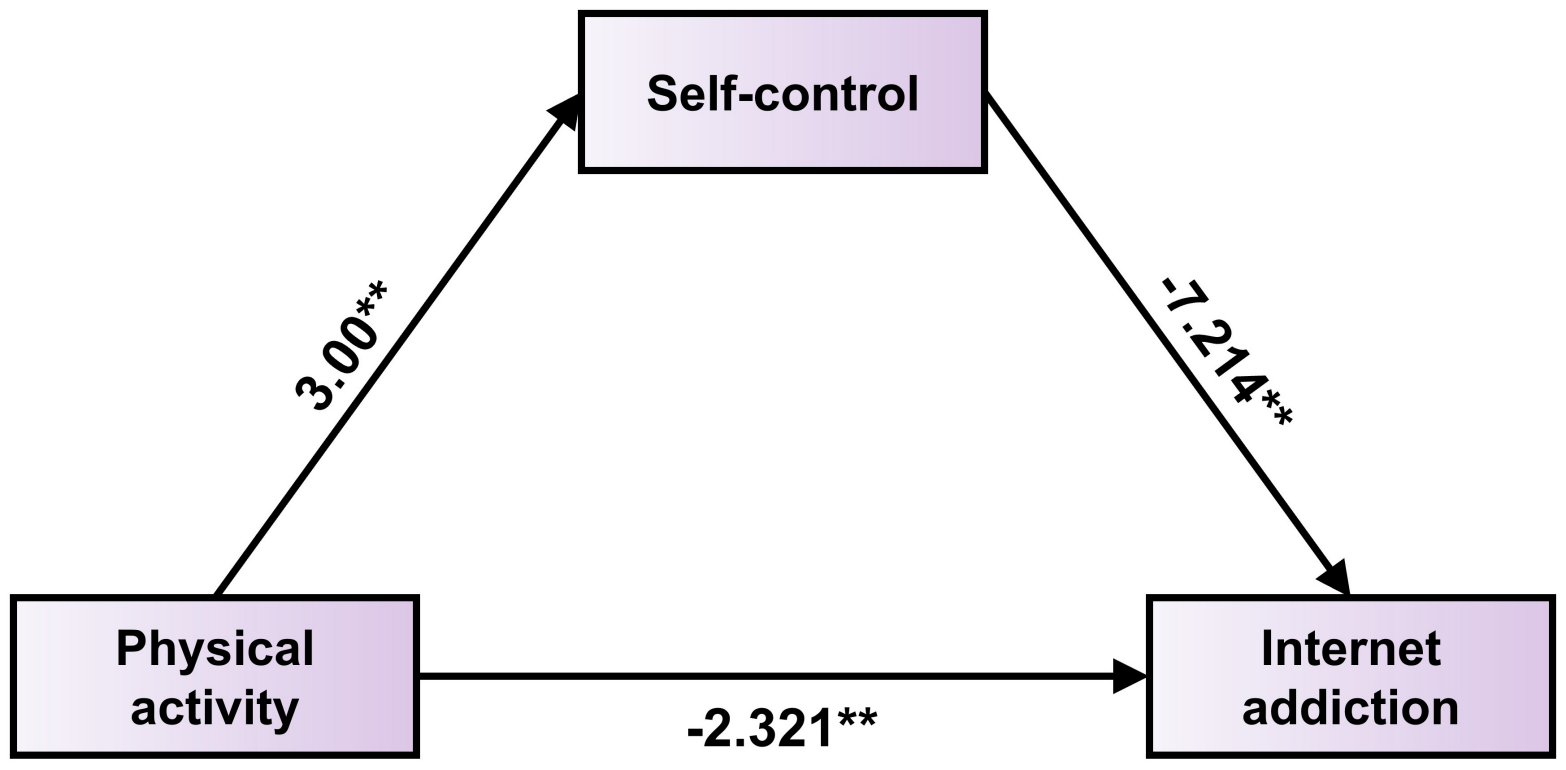
Model of the mediating role of self-control in college students' physical activity and internet addiction.

At the same time, by using the Bootstrap method in the Process macro program, combined with the stepwise test method, model 4 was used for mediation analysis. The results show that the 95% confidence intervals of *a, b, a* ^*^ *b*, *C*′, and *C* did not include 0, indicating that the effects of these paths were significant. The significant effect of physical activity on self-control indicates a direct relationship between physical activity and self-control among college students; the significant effect of self-control on internet addiction indicates a direct relationship between self-control and internet addiction among college students; the overall effect of physical activity on internet addiction is significant, indicating a direct relationship between physical activity and internet addiction among college students; the mediating effect of self-control between physical activity and internet addiction is significant, with a 95% confidence interval of (−0.085, −0.013). The results show that *a* and *b* were significant, *c*′ was not significant, and the 95% confidence interval of *a* ^*^ *b* did not include 0, indicating that the mediating effect of self-control on physical activity and internet addiction in college students is completely mediated. Refer to Table 9 and Figure 1.

**Table 9:** Test of the mediating effect of self–control on college students' physical activity and internet addiction.

**Route**	**Effect value**	**95% confidence interval**		**T statistic**	***p*–value**	**Conclusion**
		**Lower limit**	**Upper limit**			
*a*	0.006	0.002	0.009	3	0.003	Complete intermediary
*b*	−0.283	−0.36	−0.206	−7.214	0	
a^*^b	−0.002	−0.085	−0.013	−0.088	0.93	
*c′*	−0.002	−0.005	0.001	−1.426	0.154	
*C*	−0.004	−0.007	−0.001	−2.321	0.021	

a^*^b physical activity intensity (total) min/WK = self-control total score = internet addiction total score; Physical activity intensity (total) min/WK = Total self-control score; The total score of self-control is greater than the total score of internet addiction; C' Physical activity intensity (total) min/WK = Total score of internet addiction; C Physical activity intensity (total) min/WK = Total score of internet addiction.

## 5 Discussion

### 5.1 Positive guidance is needed for college students' internet addiction status

The results show that potential internet addicts (≥58 points) among college students accounted for 51.59% of the total, and internet addicts (≥68 points) accounted for 36.52% of the total. A large proportion of those diagnosed as potential internet addicts and internet addicts participated in this study. Among them, there was no significant gender difference in the degree of internet addiction tendency between boys and girls. Previous studies on internet addiction have also looked extensively at gender differences, with different conclusions based on different topics, research methods and scales used (Peisheng et al., 2022; Ha and Hwang, 2014). As per the 51st China Internet Report, China's per capita online time per week is 26.7 h. Types of Internet use include basic applications, business transactions, online entertainment, and social services. The proportion of Internet users between the ages of 10 and 29 is as high as 28.5% (China Internet Information Center, 2023). Internet use tends to be long in average time per week and rich in types of use. Research has shown that gender is not significant regarding online experience, frequency, and duration of use (Teong and Ang, 2016). A higher level of internet addiction has been observed among male subjects in relation to “online games,” “wealth management and stock speculation,” and among female subjects in relation to “shopping consumption,” “WeChat and Weibo,” and other types (Bi, 2016). Behavioral psychology posits that the greater the intensity of environmental stimuli, the higher the probability of behavioral occurrence (Qihong, 2010). In the event of the establishment of a regular and orderly correspondence between behavior and stimuli, individuals are likely to develop behavioral habits and seek corresponding stimuli on a regular basis. The characteristics of network freedom, real-time, exchange, sharing, and openness enable the realization of college students' cognitive, emotional, self-actualization, compensation, and release needs to be realized in the online world. In accordance with Maslow's hierarchy of needs, the internet serves as a means for college students to fulfill their yearnings for belonging, love, respect, and self-actualisation in a cost-effective, convenient, and secure manner. Consequently, the duration and frequency of internet usage become regular and orderly, giving rise to patterns and habits in this behavior and stimulation. It is noteworthy that the present survey is conducted in the post-pandemic period. In comparison with the learning, living and socializing experiences of the past 3 years in offline settings, the present study has revealed that the adoption of home-based online learning, living and socializing has led to a structured and consistent shift in the types of online use between male and female students. This phenomenon has the potential to intensify the prevalence of internet addiction behavior.

However, it should be noted that college students are a transitional phase from their role as students to that of social individuals. The transition is accompanied by a host of challenges, including but not limited to immaturity in both body and mind, a dearth of network literacy, and the pervasive influence of the Internet. These factors, among others, render college students particularly vulnerable to Internet addiction, fraudulent activities, substandard guidance, and illegal infringement. In light of these challenges, it is imperative for stakeholders to harness the transformative potential of network culture and new technologies for college students, create a good Internet ecology, and enable college students to participate in the construction of Internet ecology safely and effectively.

### 5.2 The significant negative impact of maternal education on college students' internet addiction

In the stepwise regression analysis of the relationship between physical activity and internet addiction among college students, it was found that the education level of the mother has a significant impact on internet addiction among college students. The mother's education level has a highly significant negative impact on internet addiction among college students, that is, the higher the education level of the mother, the lighter the tendency of internet addiction among college students.

The attachment theory of British psychoanalyst Bowlby (1969) posits that the initial relationship between a child and their primary caregiver constitutes the starting point of all relationships, especially the way the child establishes an attachment relationship with their mother (Wansen, 2010). In the event of emotional impulses related to attachment being obstructed during the development of intimate relationships, an individual may manifest behavioral and psychological disorders (Floros et al., 2012). Consequently, if the typical attachment process of college students is impeded during their developmental phase, they will resort to alternative methods to achieve attachment transfer or compensation. Internet addiction can be regarded as a compensatory demand.

Furthermore, the impact of parenting type on children is indirect, while the impact of parenting style is direct and discretionary. A close relationship exists between different parenting types and styles and adolescent behavior (Pandeng, 2014).

Research has indicated a correlation between parents' parenting styles and their children's internet addiction, particularly in terms of cognitive ability. Improper parenting styles have been found to be a significant contributing factor to their children's internet addiction. The extent to which mothers employ excessive interference, overprotection, rejection, denial, and preference in their parenting styles is positively associated with the level of internet addiction among college students (Jia, 2022; Siomos et al., 2008). The adoption of overprotective parenting styles has been demonstrated to engender limitations in the domains of self-expression and the development of independence, in addition to a diminution in problem-solving skills (Salmin et al., 2021). This phenomenon has been shown to increase the risk of Internet addiction and compromise the ability to self-regulate (Chupradit et al., 2022). In a recent cross-sectional study, overprotective parenting styles were found to be significantly associated with increased anxiety, depression, and stress among college students (La Rosa and Ching, 2024). This finding suggests a potential link between specific parenting styles and the development of Internet addiction problems, as well as the potential for these problems to contribute to the onset of psychological disorders in college students. As indicated by the extant literature, a mother's higher level of education has been shown to create a more conducive domestic environment (Chi et al., 2016). Mothers with a higher level of education are more likely to adopt positive parenting styles, which have been shown to foster children's self-discipline, effective emotional management, and the development of healthy interpersonal relationships, thereby reducing the risk of Internet addiction (Zulfiqar and Khan, 2021). It has been demonstrated that mothers who have received a higher level of education tend to continuously pursue personal growth and learning, thereby setting an example for their children (Kalil et al., 2012). The objective is to guide children in the development of correct values and attitudes toward life. Mothers with a high level of education are also more likely to prioritize the provision of a conducive educational environment and the cultivation of hobbies for their children. This approach assists children in identifying more meaningful activities as an alternative to online entertainment.

Therefore, it can be concluded that the higher the level of education of parents, especially the level of education of mothers, the more they are able to establish a positive relationship with their children, use appropriate communication methods (Song, 2023), set an example for their children and provide a wolvish upbringing and education environment. These factors subtly influence their children's physiological and psychological changes toward positive changes, thus reducing the tendency of Internet addiction.

### 5.3 Significant negative impact of physical activity on college students' internet addiction

An analysis of physical activities of varying intensities among college students revealed significant differences between male and female students in high-intensity and low-intensity physical activities. Male students participating in high-intensity physical activities were find to be significantly higher than their female counterparts, while male students participating in low-intensity physical activities were significantly lower than female students. In terms of total physical activity intensity, boys were found to be significantly lower than girls. This disparity could be attributed to the tendency for both sexes to engage in moderate-to-low intensity physical activities, such as walking, Tai Chi, and other low-intensity pursuits. It is noteworthy that the time allocated and frequency of participation in these activities exceeds that of high-intensity physical activities, such as basketball, within a week. While acknowledging the existence of gender differences, it is crucial to recognize that these are social constructs, influenced by gender role stereotypes that can significantly impact individuals' goals, expectations, values, and real-life social experiences. Within the domain of physical activity and competitive sports, gender roles are frequently employed to differentiate between boys and girls. Boys are perceived to be better suited to high-intensity, competitive physical activities, while girls are expected to participate in low-intensity, more flexible activities. This stereotypical expectation exerts a significant influence on individuals' goals, expectations, values, and actual social experiences, resulting in disparities in the intensity of physical activity between boys and girls. Societal expectations of males typically include being strong, courageous, and competitive, which makes boys more inclined to choose high-intensity physical activity to meet these expectations. Conversely, females are expected to embody qualities of gentleness and grace, and thus, low-intensity physical activities are more aligned with these social expectations. During adolescence, these gender role expectations can influence the intensity of physical activity and the selection of competitive sports for both genders.

In the analysis of the correlation between physical activity and internet addiction among college students, it was found that there is a significant negative correlation was found between total physical activity and internet addiction. This indicates that college students who engage in a wide range of physical activities over an extended period each week demonstrate a reduced propensity for internet addictionthis, finding aligns with the conclusions drawn in earlier research (Du et al., 2023).

The present study established a significant negative correlation between high-intensity physical activity (*P* < 0.01), low-intensity physical activity (*P* < 0.05), and internet addiction. In the stepwise regression analysis of the relationship between physical activity and internet addiction among college students, it was found that high-intensity physical activity (*P* < 0.01) had a significant negative impact on internet addiction among college students. The consequences of excessive internet use by college students can be detrimental to their wellbeing, potentially leading to adverse physical, psychological, and mental health outcomes, including aberrant eating habits, sleep disturbances, and reduced levels of sedentary physical activity. These consequences may also include increased feelings of loneliness and depression, reduced social engagement and satisfaction, the onset of neurological disorders, and a decline in basic interpersonal skills (Bayat et al., 2023). Research has demonstrated that individuals can ameliorate the deleterious effects of internet addiction through exercise. The underlying mechanism of this phenomenon involves the following: first, exercise elevates core body temperature and reduces muscle tension, thereby alleviating symptoms of anxiety and stress; secondly, the increase in catecholamines induced by exercise can regulate the cardiovascular and respiratory systems, control emotions, and affect attention and memory; and thirdly, the pleasure derived from endorphins produced by exercise is characterized by its prolonged duration. It has been demonstrated that exercise can enhance the specific structure and function of the central nervous system, thereby bidirectionally regulating dopamine and its receptors. This, in turn, is beneficial to human health and can effectively address the problematic behavior exhibited by adolescents with internet addiction (Siomos et al., 2008; Jun et al., 2012).

A 16-week Tai Chi intervention study was conducted on college students with internet addiction, and it was found that Tai Chi exercise can have a positive effect on the treatment of internet addiction in such students (Cuiying and Guofan, 2017). In a separate study on the impact of basketball and Baduanjin on internet addiction, it was determined that the basketball group, which engaged in high-intensity exercise, exhibited a more substantial impact in comparison to the Baduanjin group, which engaged in low-intensity activity. Nevertheless, it is noteworthy that both sporting programmes have the capacity to reduce the degree of internet addiction and promote physical and mental health in college students, albeit through different influencing mechanisms (Xiao et al., 2021). It is evident that when utilizing sports as a means to address and intervene in the issue of internet addiction among college students, it is imperative to select exercise programs that are tailored to their age-related characteristics, individual differences, and interests. For individuals grappling with internet addiction, in diverse physical activities and their corresponding varying time commitments necessitates the establishment of new lifestyles and habits, a process that is inherently intricate and necessitates the formulation of scientific exercise prescriptions tailored to specific practical circumstances.

### 5.4 Self-control fully mediates physical activity and internet addiction among college students

In this study, we looked at how self-control works as a middleman to see how exercise and internet addiction are connected. The results show that self-control had a big effect on this relationship. This means that physical activity has a direct effect on college students' internet addiction, and self-control is a key part of this process. Internet addiction among college students is a complex phenomenon influenced by a variety of factors. Self-control, acting as a mediating variable, can assist researchers in understanding the formation process of internet addiction and furnish a theoretical basis for the prevention and intervention of Internet addiction. To illustrate, dual-systems theory of self-control elucidates the internal mechanism of internet addiction.

The theory of self-control suggests that Self-control is the result of the combined action of two systems within the brain: the impulse system and the self-control system. The impulse system processes information automatically, without us being aware of it, and without using psychological resources. This means that when we try to do things like control our internet use, it can be hard to do so. Internet addiction in college students may be attributable to an impulsive system characterized by the presence of numerous internet-related behavioral schemas, and a control system that is inadequate in its capacity to generate strategies to inhibit internet use. So, to get better at controlling yourself, you need to be able to resist impulses. Engaging in exercise training can help with this in different aspects (Du et al., 2023).

The important things that can help people with internet addiction through exercise are how much exercise. The important factors that can help individuals with internet addiction through exercise include the amount of exercise they do, how hard they try, how different the exercises are, and how long they exercise for. Different types and levels of exercise have been shown to enhance self-control (Commodari et al., 2024). Exercise can make you feel good, improve your fitness, enhance your self-perception, give you more willpower and improve your self-control. It can also help college students get better at controlling themselves. The greater the self-control college students have, the less likely they are to be addicted to the internet (Bayat et al., 2023).

Therefore, in accordance with the dual systems theory of self-control, it is possible to intervene in college students' Internet addiction by improving the functioning of the control system and suppressing the influence of the impulsive system. The following intervention strategies can be adopted:

- Pre-intervention preparations: a comprehensive assessment of the target group, based on the assessment results, and the determination of goals according to the SMART principle (Elliott et al., 2022).
- Using positive thinking training to help students enhance their self-awareness and self-control, as well as to help relieve stress and anxiety, such as the correct use of the Internet and the benefits of physical activity.
- Use behavioral therapy to help students change bad behavioral habits. For example, mechanisms of rewards and punishments can be used to encourage students to use exercise tracking software, or games to increase physical activity and reduce internet use.
- Improve the functioning of the control system through cognitive therapy, cultivate alternative behaviors by adjusting the environment to achieve the impact of inhibiting the impulsive system, such as restricting the time of Internet use while increasing team building and communication training to enhance the participants' sense of teamwork and interpersonal relationship handling skills (Shuang, 2024). Individualized training. For example, for participants with anxiety, walking, tai chi, yoga, etc. can be chosen to help them relieve anxiety (Bayat et al., 2023).

## 6 Conclusion

Further research is needed on the relationship between physical activity and internet addiction both domestically and internationally, especially in terms of studying the underlying mechanisms of physical activity on internet addiction, which is still relatively scarce. This article uses the International Physical Activity Questionnaire (IPAQ) Short Volume, Chinese Internet Addiction Scale (CIAS-R), and Chinese Self Control Scale (SCS) as measurement tools, selects 471 college students as survey subjects, and tests the mediating effect of self-control on physical activity and internet addiction among college students, in order to provide reference for preventing and intervening in the deepening of college students' tendency toward internet addiction.

Firstly, it is crucial for all parties to respect college students' ability to challenge traditional gender roles, opting to engage in physical activities that align with their individual interests. Female students should be encouraged to participate in a diverse array of challenging physical activities, with a focus on ensuring ample opportunities for engagement in competitive sports. Secondly, it is vital for college students to prioritize long-term physical activities, characterized by regularity and consistency in terms of time, frequency, and intensity. This approach fosters habits that not only reduce the prevalence of internet addiction but also enhance an individual's capacity for self-control. Moreover, it is crucial for college students to enhance their self-control abilities through engaging in physical activities of varying intensity to effectively address the issue of Internet addiction, thereby providing valuable insights into the interplay between physical activity and individual psychology and behavior. In exploring the relationship between physical activity and internet addiction among college students, it is essential to consider family factors, particularly maternal parenting styles, to ensure the provision of targeted intervention and counseling.

Finally, the finding that 'self-control fully mediates the relationship between college students' physical activity and internet addiction' further enriches the dual-systems theory of self-control, providing novel perspectives and evidence for a deeper understanding of the aforementioned theory. Concurrently, the theory also provides new ideas and methods for preventing college students' Internet addiction and improving their physical activity levels.

Theoretical contribution: the present study provides new theoretical perspectives and measurement tools for the interdisciplinary field of sport and psychology, thereby contributing to an in-depth understanding of the nature and influencing factors of Internet addiction. Practical application: it provides a basis for developing targeted interventions.

## 7 Limitations

The present study did not include restructured data, which would have facilitated the demonstration of the relevant effects of differing educational levels and geographic locations. Future research could focus on the differences in self-control and behavioral habits among students with different majors, academic achievements, and educational backgrounds.

At the same time, there may be differences in lifestyle and environment among populations in different regions of China, and these differences may affect the generalizability of the findings. In future studies, the representativeness of these two factors in the study can be increased through stratified sampling by education level and different regions. In different cultural contexts, the cultural tradition in the central region focuses on family and social relationships, and students may be constrained by family and society when using the Internet. In the western region, where cultural traditions focus more on nature and traditional lifestyles, students may rely relatively less on the Internet. However, the increasing ubiquity of the internet in these regions has the potential to influence students' lives, possibly increasing the risk of Internet addiction. Future studies should investigate the possible differences and reasons for such differences in different cultural contexts.

In terms of individual factors, future research could also consider individual interest, motivation, self-esteem, self-confidence, self-efficacy, and other factors to gain a more comprehensive understanding of the differences in physical activity between male and female students and the relationship with Internet addiction.

Expanding the sample size is crucial in future research. However, this requires more resource input, higher cost, longer research time, etc. Consequently, it is imperative to methodically consider various factors and formulate a pragmatic research programme to ensure the viability and efficacy of the study.

## Data Availability

The original contributions presented in the study are included in the article/supplementary material, further inquiries can be directed to the corresponding author.
